# Gut microbiota-derived butyrate enhances exercise-induced bone mineral density in humans

**DOI:** 10.1016/j.mbm.2025.100124

**Published:** 2025-03-05

**Authors:** Xiangya Dou, Pengyu Fu, Yuting Zhang, Yiwen Zhang, Kaiting Ning, Baoqiang Yang, Xuezhou Yang, Yinbo Niu, Dong-En Wang, Huiyun Xu

**Affiliations:** a School of Life Sciences, Northwestern Polytechnical University, Xi’an, China; b Department of Sports, Northwestern Polytechnical University, Xi’an, China

**Keywords:** BMD, Exercise, Mechanical stimulus, Gut-bone axis

## Abstract

Bone Mineral Density (BMD) is one of the primary markers of bone health. Exercise provides constant mechanical stress to bone, which in turn increases BMD. Gut-bone axis is considered to play an important role in the regulation of exercise on bone. Also, the metabolites of gut microbiota (GM), especially short-chain fatty acids (SCFAs), are thought to be involved in the progress. In this study, by analyzing serum and GM from humans with low and high BMD, we found that exercise indeed enhanced BMD, and butyrate secreted from GM was involved in the regulation.

## Graphical abstract

**Figure:**
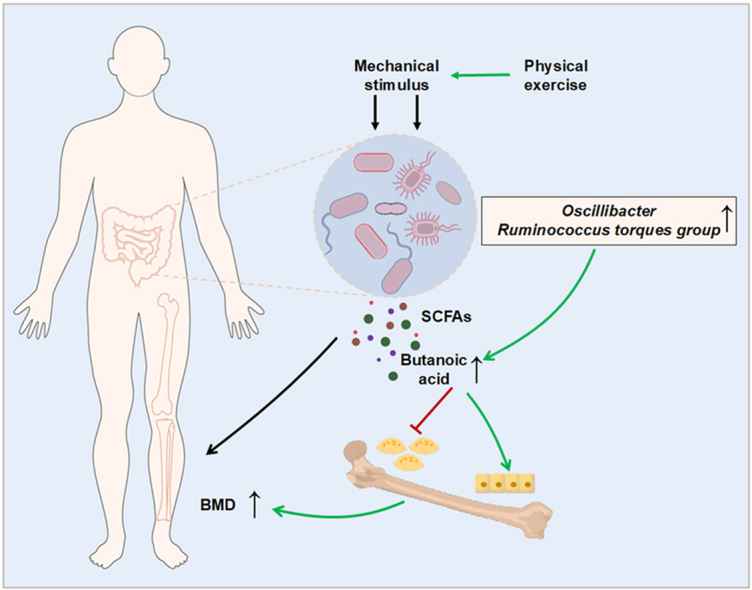

## 1 Introduction

Osteoporosis and osteopenia, mainly presenting as diminished bone mineral density (BMD), result in bone fracture and disability in the elderly and sedentary populations and have become serious problems for the public’s health.^1^^,^^2^ Bone is constantly and dynamically remodeled through osteoblast-mediated bone formation and osteoclast-mediated bone resorption. Among the various factors influencing bone quality, mechanical stimulation is crucial in maintaining bone homeostasis and function. Previous studies have shown that physical exercise provides muscle tension, ground reaction forces and impact forces for bone, which is an efficacious method for enhancing BMD, with even low-intensity spontaneous recreational activities exhibiting beneficial effects.^3^^,^^4^

Gut microbiota (GM) influences many physiological processes, including bone homeostasis. In Chinese Han youth, the α diversity of GM in osteoporosis was decreased.^5^ GM plays a vital role in maintaining bone health through various mechanisms, including influencing gut barrier function, altering the absorption of nutrients, regulating the immune system and producing beneficial metabolites, forming a complex gut-bone axis. In detail, glucocorticoid-induced or antibiotic-induced dysbiosis of GM causes bone loss via gut barrier dysfunction;^6^^,^^7^ the supplementation of probiotics increases the absorption of minerals beneficial to bone development, such as calcium and phosphate, promoting bone formation.^8^ Also, GM regulates the development and differentiation of immune cells; depletion of GM leads to bone loss through modulating lymphocyte activity.^9^^,^^10^ What’s more, GM utilizes indigestible carbohydrates to produce beneficial metabolites, such as short-chain fatty acids (SCFAs), enhancing bone formation.^11^

Our previous and other studies have shown that bone cells directly perceive and respond to mechanical stimuli, promoting osteogenic differentiation and inhibiting osteoclast differentiation.^12^, ^13^, ^14^ Exercise-induced regulation of the bone environment also occurs through cytokine paracrine; appropriate exercise can reduce osteoclast differentiation caused by proinflammatory factors and increase osteogenic differentiation induced by myogenic factor irisin^15^^,^^16^. Meanwhile, exogenous stimuli such as exercise change the composition of GM,^17^ and the absence of GM has been shown to impair the mechanical response of tibia in mice.^18^ Gut-bone axis appears to also mediate the beneficial effects of exercise on bone health. In addition, lipid metabolism and immune homeostasis are closely related to bone homeostasis,^19^^,^^20^ while exercise and probiotic supplementation improve metabolic homeostasis and immune homeostasis.^21^, ^22^, ^23^ The gut-bone axis may also influence the two processes.

SCFAs are recognized as crucial mediators in the gut-bone signaling axis. SCFAs are metabolic byproducts produced by the GM through the fermentation of indigestible fibers in food, including acetate (C2), propionate (C3), butyrate (C4), pentanoate (C5), and hexanoate (C6). They can be utilized as energy sources or incorporated into various metabolic pathways.^24^ SCFAs supplementation, mainly C2 and C3, has been demonstrated to enhance bone mass by improving osteoblastic differentiation.^25^ The supplement of C3 and C4 also inhibits osteoclast differentiation and mitigates bone loss caused by menopause and inflammation.^26^ SCFAs may be involved as crucial mediators in the process of bone remodeling.

Previous studies have illustrated the interaction between bone and gut, with most of them focused on aging-induced osteoporosis, especially in post-menopausal women.^27^, ^28^, ^29^ However, little information exists regarding healthy individuals. Limited reports point out that a robust causal relationship between GM and BMD exists throughout all stages of life; the diversity of GM phyla associated with BMD depicts relatively stable patterns between ages 0 and 45.^30^ While the α diversity of GM in osteoporosis patients is lower than in healthy persons among Chinese Han youth.^5^ In this study, we divided healthy participants into low bone mineral density (LBMD) and high bone mineral density (HBMD) groups to investigate the relationship among BMD, exercise, and GM. Our research confirms that physical exercise effectively modifies GM composition, which may subsequently enhance the production of SCFAs and ultimately increase BMD.

## 2 Materials and methods

### 2.1 Study participants and grouping

In this study, eighteen volunteers aged 18–25 years with good physical condition were recruited, and volunteers were excluded if they: (1) had a history of bone diseases; (2) had taken antibiotics or supplements such as probiotics in the last 6 months; or (3) were suffering from gastrointestinal diseases such as diarrhea and gastroenteritis. Twelve participants were included in the study. BMD at the left calcaneus was scanned by an ​ultrasonic bone densitometer (OsteoPro Smart), with the age and sex of participants inputted into the quantitative ultrasound system before the test. According to their BMD, participants were divided into two groups: LBMD group and HBMD group. All the procedures were approved by the Ethics Committee of Northwestern Polytechnical University (Approval number: 202302052), and we obtained informed consent from all of the participants.

### 2.2 Analysis of blood markers

After an 8-hour fast, blood samples were drawn from the participants. A portion of blood was placed in an anticoagulant tube and immediately mixed to prevent clotting. The remaining blood samples were left at room temperature for 30 ​min, then the serum was separated by centrifugation at 3000 ​rpm for 10 ​min using a tabletop centrifuge (Thermo Fisher Scientific). Blood cell analysis was performed using an automated hematology analyzer (Sysmex XS900I). Lipid metabolic markers, including total cholesterol (TC), triglycerides (TG), high-density lipoprotein cholesterol (HDL-C) and low-density lipoprotein cholesterol (LDL-C), as well as immune markers, including immunoglobulin A (IgA), immunoglobulin G (IgG), immunoglobulin M (IgM), complement 3 (C3) and complement 4 (C4), were analyzed by a biochemical analyzer (MS-L8080) at Northwestern Polytechnical University Hospital.

### 2.3 Identification and analysis of gut microbiota

Fecal samples uncontaminated by air or urine were collected using aseptic samplers between 8 and 10 am. The samples were immediately frozen in liquid nitrogen and stored at −80°C until further analysis. The 16S rRNA gene sequences of GM in the fecal samples were analyzed according to the standard protocols by Majorbio Bio-Pharm Technology Co., Ltd. (Shanghai, China). The raw data underwent sample demultiplexing, quality control filtering, and sequence assembly, followed by noise reduction and optimization. Subsequently, the representative sequences and abundance information of the amplicon sequence variant (ASV) were obtained. A series of statistical and visual analyses were performed based on this data, including species taxonomy, community diversity, differential species analysis, correlation analysis, phylogenetic analysis and functional prediction.

### 2.4 Identification and analysis of short-chain fatty acids

SCFAs in fecal samples were detected as described previously.^31^ Briefly, 100 ​mg of fecal sample was weighed and mixed with 900 ​μL of methanol and 100 ​μL of 2-ethylbutyric acid. The mixture was homogenized at −10°C for 6 ​min and then left to stand at −20°C for 30 ​min. Then the samples were centrifuged at 13,000×g for 15 ​min at 4°C. Subsequently, 200 ​μL of the supernatant was collected, combined with 50 ​mg of anhydrous sodium sulfate, and centrifuged again at 13,000×g for 15 ​min at 4°C. A 100 ​μL supernatant was transferred to a gas chromatography vial for analysis (Agilent 6890N GC). An HP-INNOWAX (19091N-133) capillary column was used, with nitrogen as the carrier gas. The injection volume was 1 ​μL, with a split ratio of 100:1. The initial column oven temperature was set at 50°C for 2 ​min, followed by a ramp of 10°C/min to 150°C with a 3-min hold. The temperature was then increased at 20°C/min to 250°C and held for 10 ​min before finally being reduced to 100°C and held for 2 ​min. SCFAs were qualitatively and quantitatively analyzed using Agilent OpenLab ChemStation software. Standard solutions of acetic acid, propionic acid, and butyric acid were prepared in serial dilutions to generate a standard curve.

### 2.5 Exercise questionnaire

A comprehensive questionnaire about physical exercise, including exercise duration, frequency and commonly performed exercise types, was completed via self-report (Table S1). The types of exercise were divided into low-intensity exercise, moderate-intensity exercise and high-intensity exercise. According to the approximate Metabolic Equivalent of Task (MET) of each exercise provided by the American College of Sports Medicine (ACSM), we were able to estimate the weekly METs (METs/week). The weekly METs were calculated using the following formula:

METs/week ​= ​∑(Duration of activity in minutes ​× ​METs value of the activity) ​× ​Frequency per week

### 2.6 Statistical analysis

#### 2.6.1 Basic data analysis

Except pointed out, student’s t-test was used to analyze the difference between LBMD and HBMD groups. All data were analyzed by Prism 8.0, and descriptive data were presented with mean and standard deviation. The *P* value below 0.05 was considered statistically significant.

#### 2.6.2 Gut microbiota analysis

The composition of GM was analyzed by Majorbio cloud platform (https://cloud.majorbio.com). Specifically, raw sequence data of 16S-rRNA-encoding genes were computationally processed by using the QIIME platform, the data were annotated by the Silva database. The alpha-diversity was analyzed and visualized with Mothur. The beta-diversity analysis was based on Bray–Curtis distance and ANOSIM for significance testing, and the group differences were visualized with Principal Coordination Analysis (PCoA) in Qiime2. All the other diversity analyses were conducted using R (version 3.3.1) and Python (version 2.7). The Gut Microbiome Health Index (GMHI) was used to assess the robustness of health status by comparing the abundance of GM between two groups. GMHI test between the two groups of GM was analyzed by Wilcoxon rank sum test. The linear discriminant analysis (LDA) effect size (LEfSe) was performed (LDA score greater than 3, *P* ​< ​0.05) to identify the significantly abundant taxa of bacteria. The thermal correlation analysis was performed to analyze the correlation between environmental factors and the enrichment of differential gut microbiota (DGM) and was based on Spearman. The Mantel-test analysis was performed to analyze the coefficient between the matrix of differential microbial communities and environmental factors and was based on Bray–Curtis distance and Spearman. Moreover, the linear regression analysis accentuated the influence of a single environmental factor on the diversity of sample species and was also based on Bray–Curtis distance. Finally, Spearman also was used to analyze single factor correlation network, which emphasized the correlation between species.

## 3 Results

### 3.1 Exercise improves BMD

**Fig. 1:**
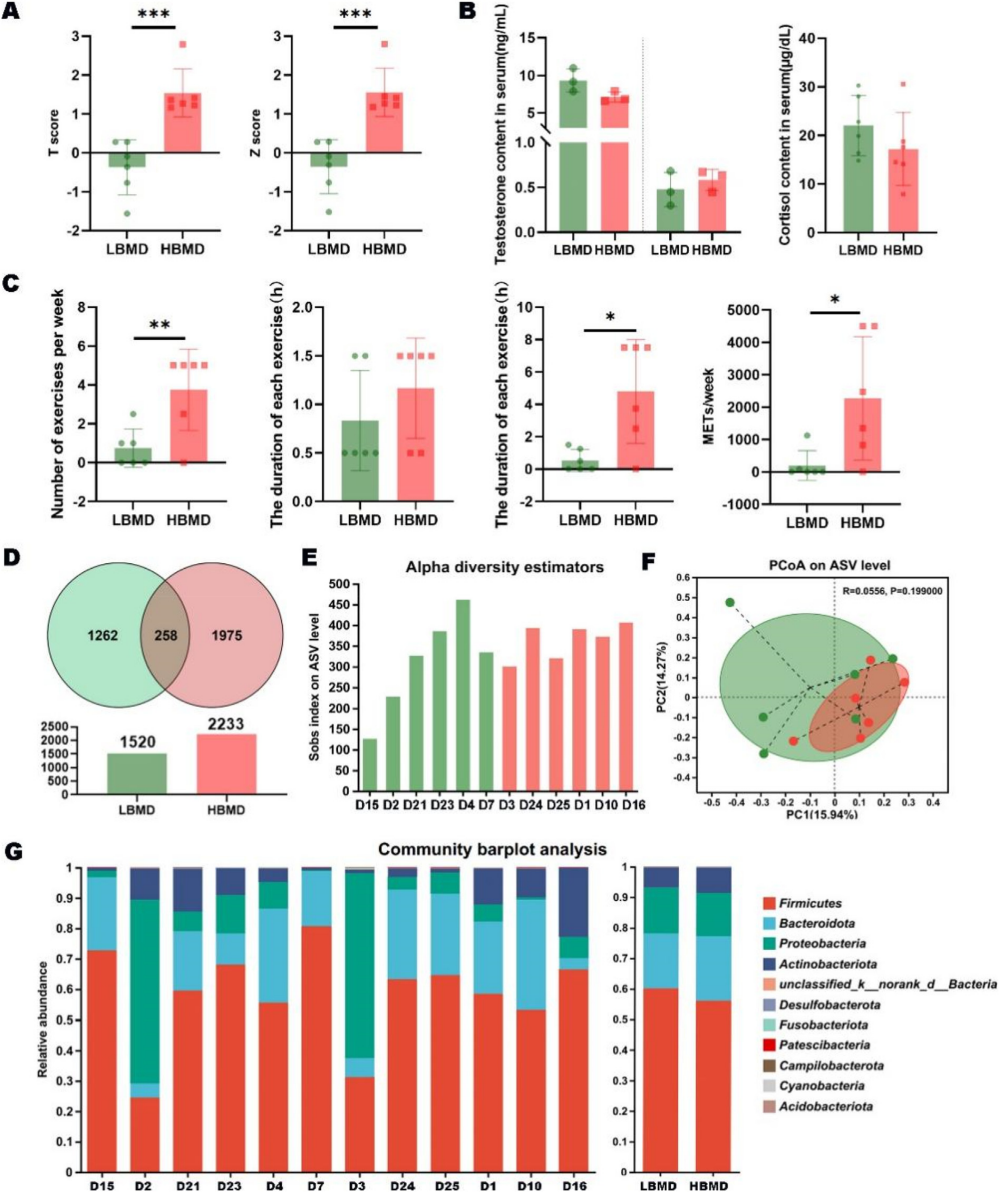
The species composition was stable at phylum level in healthy individuals. (A) The comparison analysis of BMD indexes between LBMD group and HBMD group (∗∗∗*P* ​< ​0.001). (B) The comparison analysis of cortisol and testosterone contents in the serum of the two groups. (C) The comparative analysis of exercise-related indexes between the two groups (∗∗*P* ​< ​0.01). (D) The number of ASVs measured in the two groups. (E&F) The comparison of α-diversity (sobs index) and β-diversity of GM in fecal samples. (G) The main composition of GM at the phylum level.

The statistical characteristics of the participants were summarized in Table 1. There was no significant difference in age, sex, or body mass index (BMI) between the two groups, but a highly significant difference in BMD (*P* ​< ​0.001) (Fig. 1A). Next, we investigated the reasons for the variation in BMD. Blood biochemical analysis showed no difference in cortisol or testosterone concentration between the two groups (Fig. 1B). Interestingly, analysis of the physical exercise revealed a significant difference in exercise level between the two groups. Despite a comparable duration of each exercise session, the HBMD group exhibited significantly higher exercise frequency, longer total exercise duration, and more METs (*P* ​< ​0.01) (Fig. 1C). These results underscored the important role of exercise in increasing BMD.

**Table 1:** Characteristics of the study cohorts.

	LBMD	HBMD	P value
Age	21 ​± ​1.265	21.33 ​± ​1.506	0.6867
Male/Female	3/3	3/3	>0.9999
BMI	21.03 ​± ​3.452	19.90 ​± ​1.665	0.8071

### 3.2 Exercise doesn’t affect the composition of GM at phylum level

After quality filtering of the raw data, a total of 668,778 high-quality sequences in all fecal samples were obtained. The rarefaction curves suggested comprehensive coverage of the microbes in each sample (Fig. S1A). Based on the number of ASVs measured, the species count of GM in HBMD group was increased (Fig. 1D, Fig. S1B). However, the sob diversity index showed no significant difference in community diversity of GM between LBMD and HBMD groups (Fig. 1E). Similarly, the species richness was not different between the two groups, as demonstrated by principal coordinate analysis (Fig. 1F). Furthermore, the community barplot analysis indicated that the composition of GM in healthy people was stable at the phylum level, with the most common phyla being *Firmicutes*, *Bacteroidota*, *Proteobacteria*, and *Actinobacteriota* (Fig. 1G, Figs. S1C and D).

### 3.3 Exercise improves health index, and alters the composition of GM at genus level

**Fig. 2:**
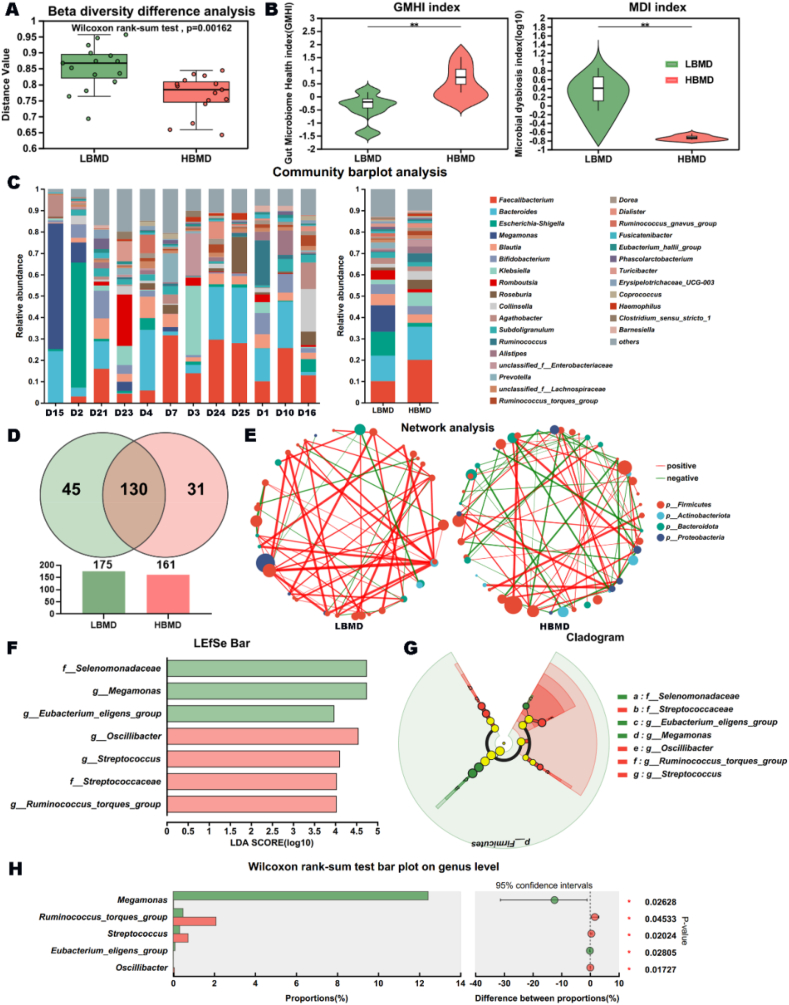
The differential microbiota between the two groups at genus level. (A) The β-diversity difference analysis between LBMD group and HBMD group. (B) The comparison analysis of GMHI index and MDI index between the two groups (∗∗*P* ​< ​0.01). (C) The main composition of GM at genus level. (D) The number of genera measured in LBMD group and HBMD group. (E) The network analysis in interspecies interactions of the two groups. (F&G) The important microbiota of the two groups. (H) The important DGMs of the two groups.

However, β-diversity difference analysis indicated that there were some differences in GM between the two groups. Specifically, compared to the LBMD group, the GMHI was significantly higher, and the microbial dysbiosis index (MDI) was significantly lower in the HBMD group (Fig. 2A and B). To investigate the alteration, we analyzed the main composition of GM at the genus level. While the GM composition was similar between the groups, the number of genera measured was decreased in HBMD group, suggesting a potential preference on GM composition within HBMD population (Fig. 2C and D). Besides, we observed a marked change in interspecies interactions according to the network analysis. There were more positive signals in LBMD group, while there were as many positive and negative signals in HBMD group, which may form a more stable community structure (Fig. 2E). To further determine the important microbiota between the two groups, we employed LEfSe analysis, which showed three key microbiota in LBMD group and four key microbiota in HBMD group at various taxonomic levels (Fig. 2F and G). Consistently, the DGM analysis of the community between the two groups further supported the significance of these key microbiota (Fig. 2H). In summary, we obtained five important DGMs at the genus level.

### 3.4 Butyrate secreted from GM involves in the promotion of exercise on BMD

**Fig. 3:**
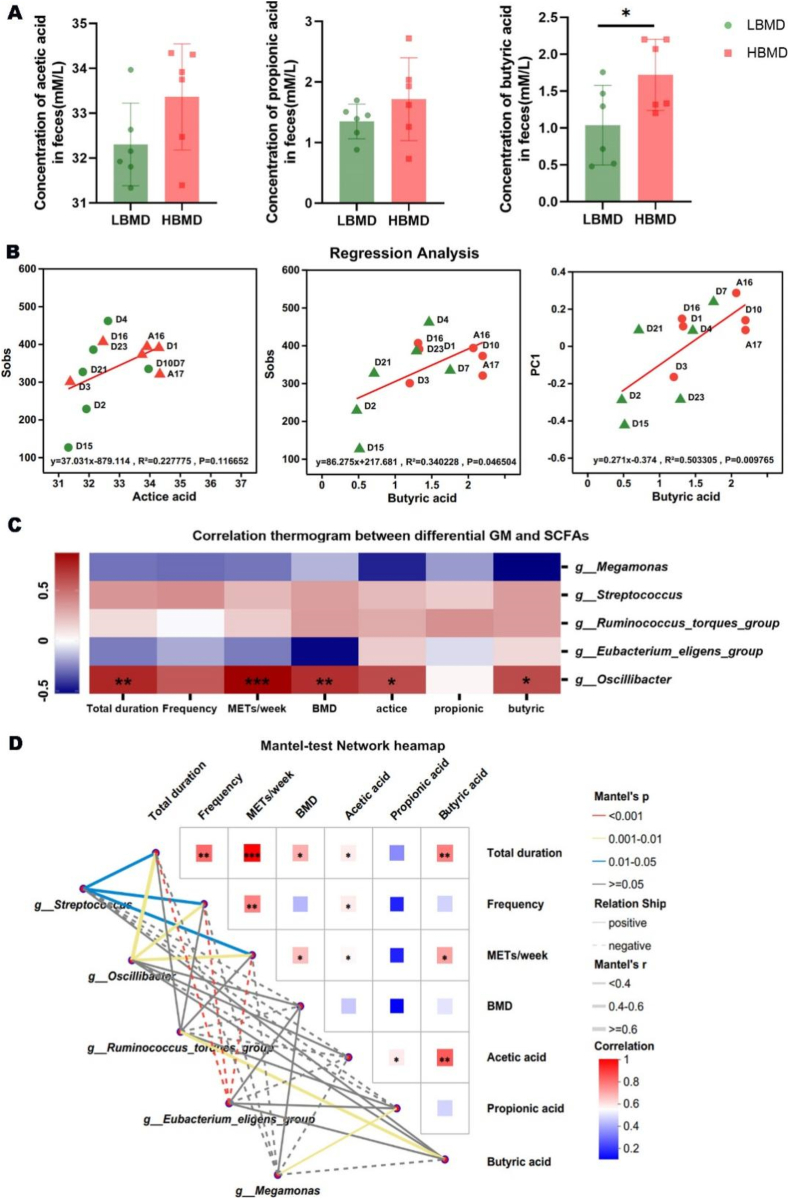
Exercise increases BMD partly by regulating butyric acid content. (A) The comparison analysis of SCFAs in LBMD group and HBMD group (∗*P* ​< ​0.05). (B) The regression analysis of SCFAs content and GM diversity or richness. (C) The heat map of single–factor correlation analysis of GM and SCFAs, GM and BMD or GM and exercise factors. (D) The mantel-test Network heatmap of GM, SCFAs, BMD and exercise factors.

It has been reported that the key DGMs, including the *Ruminococcus torques group*, *Streptococcus,* and *Oscillibacter*, are positively correlated with SCFAs level, with acetic acid and butyric acid being the primary products.^32^, ^33^, ^34^ Next, we studied the contents of acetic acid, propionic acid, and butyric acid in the fecal samples. As anticipated, the concentration of acetic acid and butyric acid in HBMD group were increased, with a particularly significant rise in butyric acid level (Fig. 3A). In addition, the abundance of butyric acid was positively correlated with the richness and diversity of GM based on the regression analysis (*P* ​< ​0.05), but the abundance of acetic acid was not significantly positively correlated with the richness of GM (Fig. 3B). The heat map from the single–factor correlation analysis indicated that butyric acid level was primarily positively correlated with the abundance of *Oscillibacter,* and the Mantel Test proved that butyric acid level was primarily positively correlated with the abundance of *R. torques group*, which highlighted the source of butyric acid. Additionally, *Oscillibacter* was strongly and positively correlated with BMD, METs, and exercise duration. The increased abundance of *Oscillibacter* and *R. torques group* in HBMD group further supported this finding, which underlined the important role of butyric acid in the regulation of physical exercise on BMD. Conversely, the *Eubacterium genes group* and *Megamonas* were decreased with longer exercise duration (Fig. 3C). Similarly, the mental-test network heatmap revealed comparable results, which extra showed a positive correlation between exercise and BMD (Fig. 3D).

### 3.5 Exercise may induce healthier lipid metabolism, without affecting immune function

**Fig. 4:**
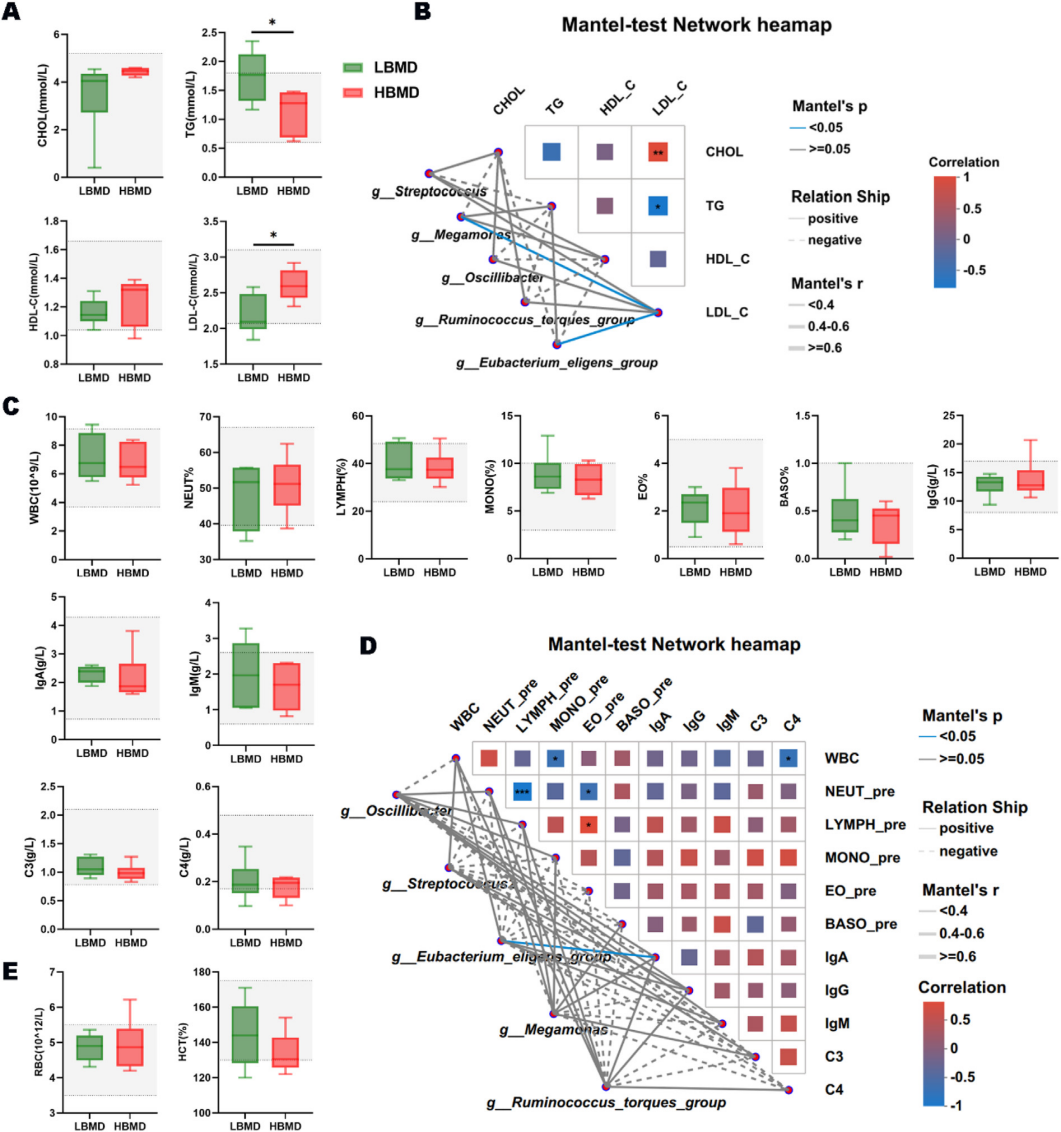
Effects of DGMs on lipid metabolism and immune ability. (A) The comparison analysis of blood lipid indexes between LBMD and HBMD group (∗*P* ​< ​0.05). (B) The mantel-test Network heatmap of GM and blood lipid indexes. (C) The comparison analysis of immune-related indexes between the two groups. (D) The mantel-test Network heatmap of GM and immune-related indexes. (E) The comparison analysis of red blood cell number and hemoglobin content between the two groups.

Studies have reported that physical exercise positively affects the metabolic system of humans.^35^ We tested the metabolic indexes of participants in both LBMD group and HBMD group. The student’s t-test indicated that the blood lipid content in the HBMD group was more aligned with health standards, although the average values were within the normal range for both groups (Fig. 4A). Meanwhile, the mantel-test analysis revealed that the abundance of *Megamonas* and *Eubacterium eligens group*, which were both decreased in HBMD, were positively correlated with low-density lipoprotein cholesterol (LDL-C), indicating that they might be key regulatory factors in the bone-gut-lipid metabolism network (Fig. 4B). Additionally, it is well established that exercise affects human immune system, which also plays an important role in the process of exercise regulating bone homeostasis.^36^ To assess the influence of DGMs on the immune system caused by exercise, we measured immune-related indexes in the peripheral blood of the two groups. As the student’s T-test showed, there was no difference between the two groups (Fig. 4C). Interestingly, the Mantel-test analysis indicated a positive association between the *E. eligens group* and immunoglobulin A (IgA) (Fig. 4D). Additionally, despite variations in physical exercise level, no significant difference was observed in blood cell number or hemoglobin content between the two groups (Fig. 4E).

## 4 Discussion

Bone is influenced by genetic and environmental factors, especially mechanical stimulation.^37^ Here, our study eliminated the potential confounding factors such as gender, age, and BMI and demonstrated that exercise, including spontaneous activity, can effectively increase BMD in healthy individuals. Previous studies have shown that exercise-induced osteogenesis is primarily mediated by bone cells, which directly sense mechanical stimuli through mechanosensitive proteins and structures, including Piezo1, Connexin 43, and primary cilia. This process promotes bone formation by activating pathways such as YAP/TAZ, Wnt/β-catenin, and ERK1/2.^38^, ^39^, ^40^ Additionally, exercise-induced osteogenesis is also indirectly regulated by cytokines from muscle, adipose tissue, and the bone itself, which influence bone cells.^38^^,^^39^^,^^41^ In recent years, the gut-bone axis has become a prominent research focus. It is reported that extracellular vesicles derived from GM in children can effectively preserve bone mass and strength.^42^ Besides, GM induces insulin-like growth factor 1 (IGF-1) production in liver and adipose tissue, which promotes bone formation and growth.^43^ Meanwhile, exercise alters GM composition and function.^17^ Ameliorating GM by exercise may also be a promoting method to facilitate bone health.

Now there is little research on the interaction between the relationship of Exercise-GM-BMD. In this study, we observed a significant difference in the composition of GM between LBMD and HBMD groups at the genus level, although overall GM structure exhibited consistency in healthy individuals. More importantly, we analyzed and emphasized the specific effects of different DGMs on BMD. Interestingly, *Oscillibacter* and *R. torques group* seem to be the main sources of butyric acid, which mediates the regulation of exercise on BMD. Previous studies have suggested that the increase of *Oscillibacter* is associated with lower cholesterol and triglyceride levels, playing a crucial role in maintaining metabolic stability.^44^^,^^45^ Besides, the abundance of *Oscillibacter* increases after exercise.^46^^,^^47^ Exercise may regulate BMD by increasing the abundance of *Oscillibacter*. In addition, the increase of *R. torques group* is related to the improvement of gut function.^48^ However, research shows that it decreases among athletes, which may be related to the high-intensity, long-term training pressure that athletes bear.^49^

*Oscillibacter* and *R. torques group* are major producers of SCFAs.^50^^,^^51^ As anticipated, our research showed that the concentration of SCFAs in HBMD group was increased with the butyric acid raised significantly. The mechanism of SCFAs regulating bone homeostasis has already been well-studied. Supplementation with SCFAs, especially propionate and butyrate, can effectively increase bone mass and prevent osteoclast differentiation caused by menopause and inflammation.^26^ At the same time, butyrate supplementation also decreases the abundance of monocytes/macrophages in bone marrow and reduces inflammation level while promoting calcium deposition in mesenchymal stem cells (MSCs), thereby facilitating fracture healing.^52^ Although Sébastien Lucas et al. argued that SCFAs did not promote osteogenesis, some studies have shown that acetate and propionate upregulate alkaline phosphatase activity, potentially because butyrate is absorbed more rapidly and extensively than acetate and propionate, thereby overshadowing the osteogenic effects of acetate.^25^ Moreover, butyrate regulates bone formation by promoting the synthesis of the osteogenic factor Wnt10b in CD8^+^ T cells, which is mediated by Treg cells.^53^ Consequently, the elevated level of butyric acid in HBMD group may be the reason for improved BMD by inhibiting bone catabolism and promoting bone anabolism.

In a word, exercise may promote BMD by regulating the composition of GM, increasing the secretion of butyric acid, and regulating the balance of bone remodeling. Regular physical exercise is effective in preventing bone loss, and the supplement of *Oscillibacter* spp. may also be an effective method to prevent bone loss. However, some strains of *Oscillibacter* possess pathogenic properties.^54^^,^^55^ Therefore, a safer approach may involve targeting its beneficial metabolic products, such as butyrate, to enhance bone health and mitigate osteoporosis risk. Butyrate supplementation could serve as a viable alternative for individuals unable to engage in regular exercise. Additionally, *R. torques group* has also made outstanding contributions to the increase of butyric acid. With an increase in sample size, *R. torques group* may also be regarded as a symbolic species of BMD. At the same time, due to the comprehensive influence of GM on human health, we also analyzed the influence of DGMs on the metabolic and immune systems. As shown, exercise-induced changes in GM have an overall positive impact on these systems. However, we acknowledge that this study has some limitations, including the use of a small sample size and a self-report questionnaire, which may affect the wide applicability of the results. More studies need to be supplemented to further prove the direct relationship between GM and BMD.

## 5 Conclusion

In summary, this study preliminarily proves that the promotion of exercise on BMD is closely related to GM. The study investigated the relationship between GM and BMD in both active and inactive healthy populations and demonstrated the crucial role of physical exercise in enhancing BMD. Findings further suggest that exercise may improve BMD by modulating the composition of GM and promoting butyric acid production. Regular physical exercise is effective in preventing bone loss, and butyrate supplementation could serve as a viable alternative for individuals unable to engage in regular exercise. This study provides convincing evidence for the new view of exercise altering GM to promote bone health and supports the potential of nutrition intervention to improve BMD, which provides a new direction for the research on the mechanism of exercise intervention to prevent bone loss.

## CRediT authorship contribution statement

**Xiangya Dou:** Writing – original draft, Visualization, Investigation, Formal analysis. **Pengyu Fu:** Methodology, Investigation. **Yuting Zhang:** Investigation. **Yiwen Zhang:** Investigation. **Kaiting Ning:** Validation, Data curation. **Baoqiang Yang:** Validation, Data curation. **Xuezhou Yang:** Validation, Data curation. **Yinbo Niu:** Project administration. **Dong-En Wang:** Project administration. **Huiyun Xu:** Writing – review & editing, Conceptualization.

## Ethical approval

This study was approved by the Ethics Committee of Northwestern Polytechnical University (Xian, China. Approval number: 202302052). All participants provided informed consent prior to data collection.

## Declaration of competing interest

The authors declare no competing interests.

## References

[1] Onizuka N., Onizuka T. (2024). Disparities in osteoporosis prevention and care: understanding gender, racial, and ethnic dynamics. Curr Rev Musculoske.

[2] Lin Z., Shi G., Liao X. (2023). Correlation between sedentary activity, physical activity and bone mineral density and fat in America: National Health and Nutrition Examination Survey, 2011-2018. Sci Rep.

[3] Manske S.L., Lorincz C.R., Zernicke R.F. (2009). Bone health: part 2, physical activity. Sports Health.

[4] Callréus M., McGuigan F., Ringsberg K., Åkesson K. (2012). Self-reported recreational exercise combining regularity and impact is necessary to maximize bone mineral density in young adult women A population-based study of 1,061 women 25 years of age. Osteoporos Int.

[5] Lai J.R., Gong L., Liu Y. (2024). Associations between gut microbiota and osteoporosis or osteopenia in a cohort of Chinese Han youth. Sci Rep.

[6] Schepper J.D., Collins F.L., Rios-Arce N.D. (2019). Probiotic *Lactobacillus reuteri* prevents post-antibiotic bone loss by reducing intestinal dysbiosis and preventing barrier disruption. J Bone Min Res.

[7] Schepper J.D., Collins F., Rios-Arce N.D. (2020). Involvement of the gut microbiota and barrier function in glucocorticoid-induced osteoporosis. J Bone Min Res.

[8] Rizzoli R. (2019). Nutritional influence on bone: role of gut microbiota. Aging Clin Exp Res.

[9] Kamada N., Seo S.U., Chen G.Y., Núñez G. (2013). Role of the gut microbiota in immunity and inflammatory disease. Nat Rev Immunol.

[10] Rios-Arce N.D., Schepper J.D., Dagenais A. (2020). Post-antibiotic gut dysbiosis-induced trabecular bone loss is dependent on lymphocytes. Bone.

[11] Grüner N., Ortlepp A.L., Mattner J. (2023). Pivotal role of intestinal microbiota and intraluminal metabolites for the maintenance of gut-bone physiology. Int J Mol Sci.

[12] Zhao D.Z., Riquelme M.A., Guda T. (2022). Connexin hemichannels with prostaglandin release in anabolic function of bone to mechanical loading. Elife.

[13] Zhou T.F., Gao B., Fan Y. (2020). Piezo1/2 mediate mechanotransduction essential for bone formation through concerted activation of NFAT-YAP1-ss-satenin. Elife.

[14] Shen B., Tasdogan A., Ubellacker J.M. (2021). A mechanosensitive peri-arteriolar niche for osteogenesis and lymphopoiesis. Nature.

[15] Cheng L.J., Khalaf A.T., Lin T.C. (2020). Exercise promotes the osteoinduction of HA/β-TCP biomaterials via the Wnt signaling pathway. Metabolites.

[16] Bao J.F., She Q.Y., Hu P.P., Jia N., Li A.Q. (2022). Irisin, a fascinating field in our times. Trends Endocrin Met.

[17] Wegierska A.E., Charitos I.A., Topi S., Potenza M.A., Montagnani M., Santacroce L. (2022). The connection between physical exercise and gut microbiota: implications for competitive Sports athletes. Sports Med.

[18] Wang D., Cai J., Pei Q.L. (2024). Gut microbial alterations in arginine metabolism determine bone mechanical adaptation. Cell Metab..

[19] Kang J., Zhao S.L., Wu X.Z., Wang C., Jiang Z.K., Wang S.X. (2023). The association of lipid metabolism with bone metabolism and the role of human traits: a Mendelian randomization study. Front Endocrinol.

[20] Fischer V., Haffner-Luntzer M. (2022). Interaction between bone and immune cells: implications for postmenopausal osteoporosis. Semin Cell Dev Biol.

[21] Li V.L., He Y., Contrepois K. (2022). An exercise-inducible metabolite that suppresses feeding and obesity. Nature.

[22] Van Hul M., Cani P.D. (2023). The gut microbiota in obesity and weight management: microbes as friends or foe?. Nat Rev Endocrinol.

[23] Martin K.S., Azzolin M., Ruas J.L. (2020). The kynurenine connection: how exercise shifts muscle tryptophan metabolism and affects energy homeostasis, the immune system, and the brain. Am J Physiol-Cell Ph.

[24] Feng B.Y., Lu J.J., Han Y.H., Han Y.G., Qiu X.K., Zeng Z.Y. (2024). The role of short-chain fatty acids in the regulation of osteoporosis: new perspectives from gut microbiota to bone health: a review. Medicine.

[25] Kondo T., Chiba T., Tousen Y. (2022). Short-chain fatty acids, acetate and propionate, directly upregulate osteoblastic differentiation. Int J Food Sci Nutr.

[26] Lucas S., Omata Y., Hofmann J. (2018). Short-chain fatty acids regulate systemic bone mass and protect from pathological bone loss. Nat Commun.

[27] Guan Z.Y., Xuanqi Z., Zhu J.X. (2023). Estrogen deficiency induces bone loss through the gut microbiota. Pharmacol Res.

[28] Lin X., Xiao H.M., Liu H.M. (2023). Gut microbiota impacts bone via Bacteroides vulgatus-valeric acid-related pathways. Nat Commun.

[29] Huidrom S., Beg M.A., Masood T. (2021). Post-menopausal osteoporosis and probiotics. Curr Drug Targets.

[30] Wang Y., Zhang X.J., Tang G.J. (2023). The causal relationship between gut microbiota and bone mineral density: a Mendelian randomization study. Front Microbiol.

[31] Zhou M.S., Zhang B., Gao Z.L. (2021). Altered diversity and composition of gut microbiota in patients with allergic rhinitis. Microb Pathogenesis.

[32] Wan F., Wang M.Y., Zhong R.Q. (2022). Supplementation with Chinese medicinal plant extracts from and mitigates colonic inflammation by regulating oxidative stress and gut microbiota in a colitis mouse model. Front Cell Infect Microbiol.

[33] Yao D., Wu M.N., Dong Y. (2022). *In vitro* fermentation of fructooligosaccharide and galactooligosaccharide and their effects on gut microbiota and SCFAs in infants. J Funct Foods.

[34] Liu B.Y., Zhang Z.G., Liu X.Q., Hu W.W., Wu W.C. (2023). Gastrointestinal fermentable polysaccharide is beneficial in alleviating loperamide-induced constipation in mice. Nutrients.

[35] Mann S., Beedie C., Jimenez A. (2014). Differential effects of aerobic exercise, resistance training and combined exercise modalities on cholesterol and the lipid profile: review, synthesis and recommendations. Sports Med.

[36] Cornish S.M., Chilibeck P.D., Candow D.G. (2020). Potential importance of immune system response to exercise on aging muscle and bone. Curr Osteoporos Rep.

[37] Wang L.J., You X.L., Zhang L.L., Zhang C.Q., Zou W.G. (2022). Mechanical regulation of bone remodeling. Bone Res.

[38] Li X., Han L., Nookaew I. (2019). Stimulation of Piezo1 by mechanical signals promotes bone anabolism. Elife.

[39] Barton W., Penney N.C., Cronin O. (2018). The microbiome of professional athletes differs from that of more sedentary subjects in composition and particularly at the functional metabolic level. Gut.

[40] Sun Y.X., Yuan Y., Wu W., Lei L., Zhang L.L. (2021). The effects of locomotion on bone marrow mesenchymal stem cell fate: insight into mechanical regulation and bone formation. Cell Biosci.

[41] Kirk B., Feehan J., Lombardi G., Duque G. (2020). Muscle, bone, and fat crosstalk: the biological role of myokines, osteokines, and adipokines. Curr Osteoporos Rep.

[42] Liu J.H., Chen C.Y., Liu Z.Z. (2021). Extracellular vesicles from child gut microbiota enter into bone to preserve bone mass and strength. Adv Sci.

[43] Yan J., Herzog J.W., Tsang K. (2016). Gut microbiota induce IGF-1 and promote bone formation and growth. Proc Natl Acad Sci USA.

[44] Li C.H., Strazar M., Mohamed A.M.T. (2024). Gut microbiome and metabolome profiling in Framingham heart study reveals cholesterol-metabolizing bacteria. Cell.

[45] Liu X.M., Tong X., Zou Y.Q. (2022). Mendelian randomization analyses support causal relationships between blood metabolites and the gut microbiome. Nat Genet.

[46] Liang R., Zhang S., Peng X.J. (2019). Characteristics of the gut microbiota in professional martial arts athletes: a comparison between different competition levels. Plos One.

[47] Wang G., Zhou H.H., Luo L. (2021). Voluntary wheel running is capable of improving cognitive function only in the young but not the middle-aged male APPSwe/PS1De9 mice. Neurochem Int.

[48] Zhao Y.C., Zhan J.G., Sun C.Y. (2024). Sishen Wan enhances intestinal barrier function via regulating endoplasmic reticulum stress to improve mice with diarrheal irritable bowel syndrome. Phytomedicine.

[49] Hintikka J.E., Munukka E., Valtonen M. (2022). Gut microbiota and serum metabolome in elite cross-country skiers: a controlled study. Metabolites.

[50] Yao Y., Yan L.J., Chen H., Wu N., Wang W.B., Wang D.S. (2020). *Cyclocarya paliurus* polysaccharides alleviate type 2 diabetic symptoms by modulating gut microbiota and short-chain fatty acids. Phytomedicine.

[51] Verhaar B.J.H., Hendriksen H.M.A., de Leeuw F.A. (2022). Gut microbiota composition is related to AD pathology. Front Immunol.

[52] Wallimann A., Magrath W., Pugliese B. (2021). Butyrate inhibits osteoclast activity and regulates systemic inflammation and bone healing in a murine osteotomy model compared to antibiotic-treated mice. Mediat Inflamm.

[53] Tyagi A.M., Yu M.C., Darby T.M. (2018). The microbial metabolite butyrate stimulates bone formation via T regulatory cell-mediated regulation of WNT10B expression. Immunity.

[54] Sydenham T.V., Arpi M., Klein K., Justesen U.S. (2014). Four cases of bacteremia caused by, a newly described species. J Clin Microbiol.

[55] Broutin L., Deroche L., Michaud A. (2020). First description of bacteremia caused by in a patient hospitalized for leg amputation. Anaerobe.

