# Supplementary material for: Gut microbiota-derived butyrate enhances exercise-induced bone mineral density in humans
Source: Mechanobiol Med. 2025 Mar 5;3(2):100124. doi: 10.1016/j.mbm.2025.100124 (PMC12067894; doi:10.1016/j.mbm.2025.100124)
Supplement: Multimedia component 2 [file mmc2.docx]

**Supplementary figure 1**


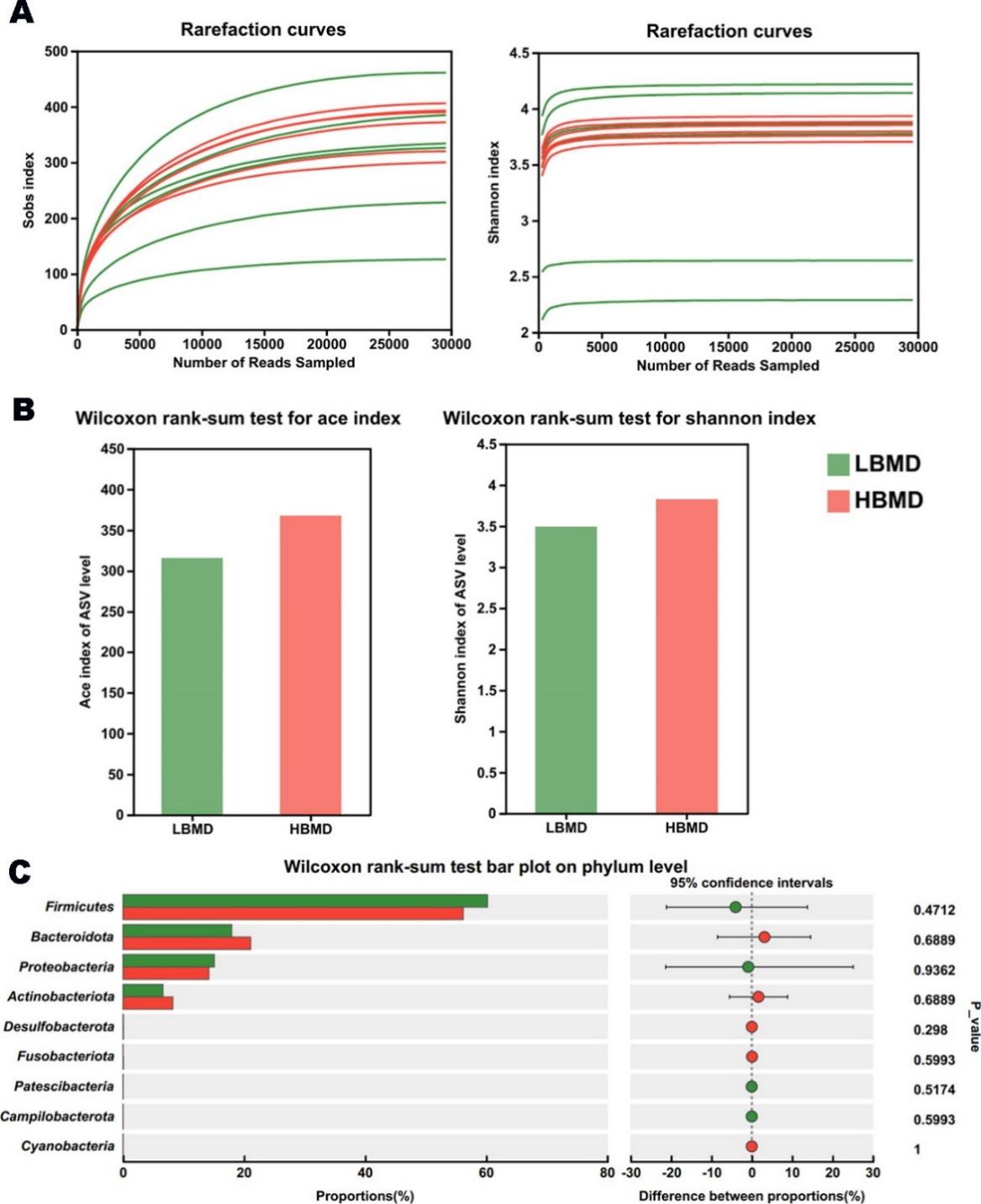


Figure S1. Characteristics of GM in LBMD and HBMD groups

(A) The rarefaction curves of the two groups at ASV level. (B) The comparison analysis on diversity and richness of GM. (C) The comparison analysis on GM between the two groups at phylum level.
